# Assembly of 1D Granular Structures from Sulfonated Polystyrene Microparticles

**DOI:** 10.3390/ma10101212

**Published:** 2017-10-21

**Authors:** Alexander Mikkelsen, Ahmet Kertmen, Khobaib Khobaib, Michal Rajňák, Juraj Kurimský, Zbigniew Rozynek

**Affiliations:** 1 Institute of Acoustics, Faculty of Physics, Adam Mickiewicz University, Umultowska 85, 61-614 Poznań, Poland; alexam@amu.edu.pl (A.M.); khobaib@amu.edu.pl (K.K.); 2 Faculty of Chemistry, Gdańsk University of Technology, Narutowicza 11/12, 80-233 Gdańsk, Poland; ahmet.kertmen@pg.gda.pl; 3 NanoBioMedical Centre, Adam Mickiewicz University, Umultowska 85, 61-614 Poznań, Poland; 4 Institute of Experimental Physics SAS, Watsonova 47, 040-01 Kosice, Slovakia; rajnak@saske.sk; 5 Faculty of Electrical Engineering and Informatics, Technical University of Košice, Letná 9, 04200 Košice, Slovakia; juraj.kurimsky@tuke.sk

**Keywords:** spherical polystyrene particles, microparticles, sulfonation, electric conductivity, dielectric constant, electric fields, field-driven assembly, 1D structures

## Abstract

Being able to systematically modify the electric properties of nano- and microparticles opens up new possibilities for the bottom-up fabrication of advanced materials such as the fabrication of one-dimensional (1D) colloidal and granular materials. Fabricating 1D structures from individual particles offers plenty of applications ranging from electronic sensors and photovoltaics to artificial flagella for hydrodynamic propulsion. In this work, we demonstrate the assembly of 1D structures composed of individual microparticles with modified electric properties, pulled out of a liquid environment into air. Polystyrene particles were modified by sulfonation for different reaction times and characterized by dielectric spectroscopy and dipolar force measurements. We found that by increasing the sulfonation time, the values of both electrical conductivity and dielectric constant of the particles increase, and that the relaxation frequency of particle electric polarization changes, causing the measured dielectric loss of the particles to shift towards higher frequencies. We attributed these results to water adsorbed at the surface of the particles. With sulfonated polystyrene particles exhibiting a range of electric properties, we showed how the electric properties of individual particles influence the formation of 1D structures. By tuning applied voltage and frequency, we were able to control the formation and dynamics of 1D structures, including chain bending and oscillation.

## 1. Introduction

The fabrication of one-dimensional (1D) colloidal and granular materials is presently a very active area of nano- and micromaterials research. Colloidal and granular 1D assemblies offer great opportunities for both fundamental studies [1,2,3] and practical applications, including applications in optoelectronics [4], photonics [5], sensors [6], photovoltaics [7], and flexible electronics [8]. The 1D assemblies can be composed of either particle groups [9,10,11] or individual particles forming particle chain-like structures [12,13,14]. 1D particle structures are typically formed along a substrate, but out-of-plane structures can be also designed, such as flexible artificial flagella or cilia [15,16,17]. Here, we research 1D structures composed of individual granular microparticles, i.e., assemblies with a thickness of a single particle.

There are many strategies to make 1D colloidal and granular assemblies, including lithography methods [18], pore-assisted assembly [19], dip-coating [20], step edge decoration [21], and field-directed assembly in electro- or magneto-rheological fluids [22,23]. The latter is generally considered to be a simple and effective approach to form particle structures. Despite some attempts to fabricate 2D particle assemblies (network structures) [24], the field-directed method is primarily used for the formation of linear structures by applying external magnetic or electric fields. 

Field-assisted organization of 1D colloidal and granular particles usually takes place in a solution [25,26] or at liquid interfaces [27,28]. Recently, we reported a route to fabricating particle chain-like structures outside liquid environments [29]. We proposed combining electric field-directed assembly with capillary effects. In short, the method requires an application of an external electric field to polarize conductive particles that are initially dispersed in a leaky dielectric liquid, such as synthetic oils. The particles are attracted toward the electrode placed right above the liquid-air interface. The attractive force acting on particles stems from dielectrophoresis. By raising the electrode, the particles are pulled out of the liquid to form chain-like structures of the desired length, thickness, and mechanical properties. In that previous research, the experiments were performed with conductive microparticles made of stainless steel or hollow glass microspheres coated with silver [29]. Such particles are very costly in comparison to polymeric materials, i.e., silver-coated particles are around 10^4^ times more expensive than polystyrene (PS) particles of similar size and polydispersity. Motivated by material cost reduction, we here investigate the possibilities of modifying PS particles and tailoring the electric properties of these particles for assembly purposes. Changing the electric properties of polymeric particles can also be useful for other purposes, such as reducing the accumulation of static electricity by increasing charge transport through materials [30,31] or enhancing the electrorheological response [32]. Tailoring the electric properties also make the particles good candidates for material to be used, for example, in conductive particle inks [33], proton exchange membranes [34], or anisotropic conducting adhesives [35].

The electric properties of PS particles can be modified by physical or chemical methods, including surface coating by conductive polymers [36,37,38], surface decorating with conductive nanoparticles [39,40,41], or surface modification by sulfonation processes [42,43,44]. As demonstrated in our recent research [42], the sulfonation of PS particles yields polymeric particles with a large range of electrical properties that are related to the degree of sulfonation (and water content) controlled by the reaction process (i.e., reaction time, temperature, or concentration of sulfuric acid). The sulfonation process can also be used to change other physical properties, such as particle hardness and stickiness, as well as properties that can be further exploited, e.g., to create composite microcapsules with different morphologies [45,46].

**Figure 1:**
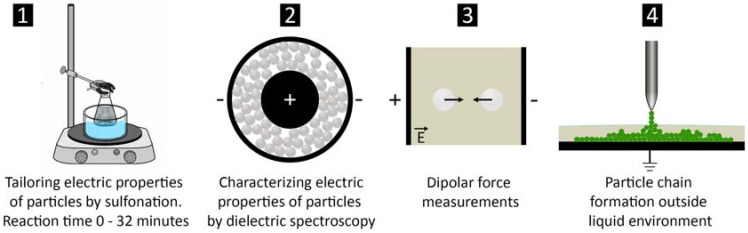
Scheme of the study. (**1**) The electric properties of polystyrene particles are tailored by sulfonating particles for different reaction times; (**2,3**) Differences in electric properties of the particles are characterized and verified by dielectric spectroscopy and dipolar force measurements; (**4**) Particle chaining from oil to air environments is investigated for pure and modified polystyrene particles.

In this work, we also investigate how sulfonation can be used to produce PS microparticles with different electrical conductivity and dielectric constants. Compared to our previous work [42], the particles here are sulfonated under different reaction conditions (concentration of sulfuric acid and reaction environment during sulfonation), and their electric properties are characterized by measuring their electric conductivities, dielectric constant, and dielectric loss values. The effect of sulfonation on the electric properties of the particles is further studied by performing dipolar-force measurements on particle pairs suspended in fluid and in applied electric fields. We then test whether sulfonated PS particles with tailored electrical properties can be used to fabricate chain-like structures outside bulk liquid through the synergetic action of dielectrophoresis and capillary effects. The study is summarized in Figure 1.

## 2. Results

We use PS particles (with a mean diameter of around 140 μm) sulfonated at incremental reaction times (1 min, 2 min, 4 min, 8 min, 16 min, and 32 min) to study the influence of the sulfonation reaction time on the final physical properties of the sulfonated PS particles, and their capabilities to form 1D structures in presence of electric fields. The pristine PS particles, from which the sulfonated particles were made, are also studied here as a reference sample.

### 2.1. Dielectric Constant and Electrical Conductivity

The dielectric constant (*ε*) and electrical conductivity (*σ*) of the PS particles were measured using an LCR meter with a frequency (*f*) range between 20 Hz and 100 kHz. The LCR meter was set to parallel capacitor mode, where the capacitance (*C*) and the dissipation factor (tan*δ*) were recorded. The dielectric constant and electrical conductivity values were calculated from the equations: *ε* = *C*/*C*_o_, *σ* = 2π*fC* tan*δ*(*l*/*A*), where *C*_o_ is the capacitance value of the empty capacitor, *l* is the separation distance between electrodes, and *A* is the effective electrode area (for details see the Methods section).

**Figure 2:**
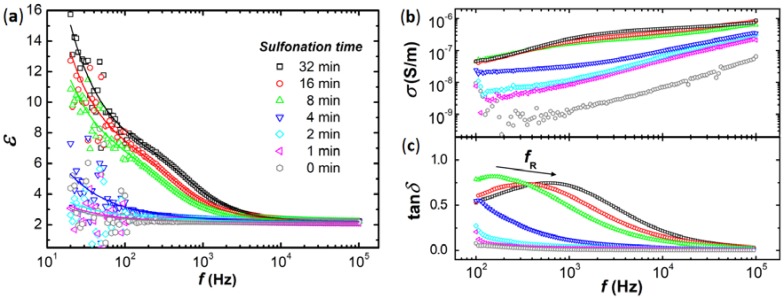
Effect of sulfonation reaction time on dielectric constant (**a**); electrical conductivity (**b**); dielectric loss factor values (**c**) of the PS particles. The dielectric constant experimental data is fitted by a combination of the Havriliak-Negami function and the Jonscher power law model (for details see the Methods section).

Figure 2 presents dielectric constant-, dielectric loss factor-, and electrical conductivity frequency spectra for the non-sulfonated PS sample and the six chemically modified PS samples at different sulfonation times. The dielectric constant values (Figure 2a) of all samples increase when the frequency of the applied electric field is decreased, and they are consistently higher for the PS particles that were modified for longer reaction times. The dielectric constant of the PS particles modified for 32 min is more than three times higher than that of the non-sulfonated PS particles (at *f* = 100 Hz).

A similar trend applies to the measured conductivity values presented in Figure 2b. The electrical conductivity values are highest for the particles sulfonated for 32 min, and are for all frequencies more than one order of magnitude higher than the conductivity values of the non-sulfonated PS particles. The electrical conductivity values are consistently higher for the PS particles modified at longer reaction times. Figure 2c shows that the relaxation frequency (*f*_R,_ frequency at which the dissipation factor attains its maximum) systematically increases for samples sulfonated for longer times: *f*_R_ = 150 Hz for the sample sulfonated for 8 min and increases to around 330 Hz and 600 Hz for PS samples sulfonated for 16 and 32 min, respectively (indicated by the arrow in Figure 2c). The change in the measured particle electric properties with sulfonation reaction time is likely due to an increase in the particle’s abilities to adsorb water at their interface (for details see the Discussion and Conclusion sections). Table 1 lists the calculated values of *ε* and *σ* at *f* = 100 Hz (the frequency that is used in experiments presented in Section 2.2 and Section 2.3). The obtained values and data trends for both the dielectric constant and electrical conductivity are similar to those obtained from our previous set of samples in Reference [42]. Table 1 also includes the estimated values of the dielectric strength ($\varepsilon_{S}-\varepsilon_{\infty }$), where $\varepsilon_{S}$ and $\varepsilon_{\infty }$ are the low and high frequency limit of the complex dielectric permittivity, $\varepsilon^{*}\left(\omega \right)$, of each sample obtained by fitting the experimental dielectric constant data to theoretical models (for details see the Methods section). Note that the dielectric constant value of the unmodified PS particles exhibits quasi constant behavior in the whole frequency range (Figure 2a), and that the measured value (2.2) is lower than expected for the PS material (~2.5). We associate this with air spaces present in the measured powder.

### 2.2. Dipolar Forces between Particles

**Figure 3:**
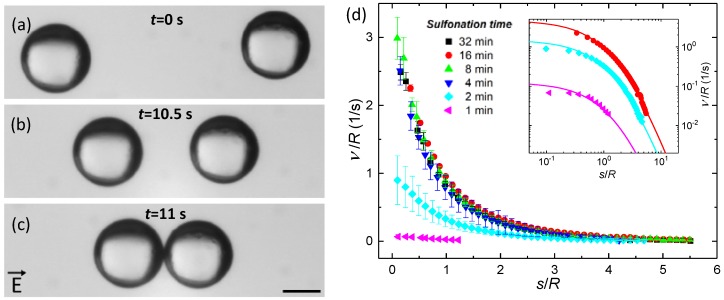
Dipolar force between sulfonated particles suspended in 100 mPa·s silicone oil. (**a**–**c**) When subjected to an AC electric field of 75 V/mm (100 Hz), the attractive dipolar force aligns and brings 140-µm polystyrene particles (sulfonated for 16 min) together. The scale bar is 100 μm, and the electric field is in the horizontal direction; (**d**) Average particle speed plotted versus average particle separation for PS particles sulfonated for 1–32 min. In the inset, the data points of particles sulfonated for 1, 2, and 16 min are fitted with a dipolar model (for details, see below). The data points for the other particles (sulfonated for 4, 8, and 32 min) overlap with the data points for PS16 (red circles) and are omitted for visual clarity.

The dielectric constant values of the PS particles can be measured quantitatively by investigating the interparticle dipolar forces between particles suspended in liquid and subjected to electric fields. Polystyrene particles sulfonated for 1–32 min were suspended in 100 mPa·s silicone oil, brought in close proximity to each another, and subjected to a 100-Hz electric field of 75 V/mm (root-mean-square (RMS), square wave). Figure 3a–c show how the attractive dipolar force between PS particles (sulfonated for 16 min) aligns and brings the particles together. It takes around 11 s to bring the particles from a separation distance of around 2 particle diameters (Figure 3a) to particle contact (Figure 3c).

Figure 3d presents the average particle speed plotted versus the particle separation distance, both normalized to R. The speed of the particles increases as the separation distance between them decreases. The particle speed is highest and similar for the PS particles sulfonated for 8–32 min, while particles sulfonated for shorter times (1–4 min) move significantly slower. 

For the two-particle system we have the following force balance: $F_{dip}\left(s\right)=-F_{drag}$, where $F_{dip}\left(s\right)$ is the dipolar force acting on the particles and $F_{drag}$ is the drag force of viscosity on the particles. For a spherical object with radius *R* moving with speed ν in a fluid with viscosity η, Stoke’s law states: $F_{drag}=6\pi \eta R\nu$. The dipolar force between two suspended particles that are subjected to a high frequency alternating current (AC) electric field of strength $E_{0}$ is given by [47]: $F_{dip}\left(s\right)=\frac{24\pi \varepsilon_{0}\varepsilon_{f}R^{6}\beta^{2}E_{0}^{2}}{{\left(2R+s\right)}^{4}}$, where *s* is the separation distance between particles (edge to edge), $\beta =\left(\varepsilon_{p}-\varepsilon_{f}\right)/\left(\varepsilon_{p}+2\varepsilon_{f}\right)$ where $\varepsilon_{p}$ and $\varepsilon_{f}$ are the particle and fluid dielectric constants, respectively, and $\varepsilon_{0}$ is the vacuum permittivity. Because we experimentally measure *s*, we find it convenient to rewrite the particle center to center distance in Reference [47] to 2*R* + *s*. Combining the equations and solving for the particle speed yields: $\nu \left(s\right)=\frac{4\varepsilon_{0}\varepsilon_{f}R^{5}\beta^{2}E_{0}^{2}}{\eta {\left(2R+s\right)}^{4}}$.

In the inset of Figure 3d, the measured normalized particle speeds have been fitted to the speed equation above where the distances (R and s) were normalized to R. The data fitting procedure yields quantitative β and $\varepsilon_{p}$ values for the different particles, and these are listed in Table 2. 

### 2.3. Electric Field-Assisted Formation of Granular Chains

**Figure 4:**
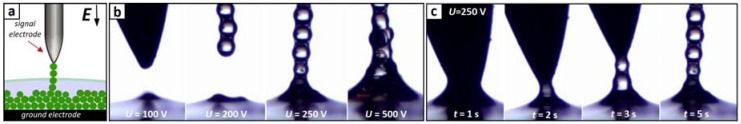
(**a**) A schematic representation of the fabrication process of particle chains; (**b**) Experiments on granular particle chain fabrication. The strength of the applied voltage (f = 100 Hz, square wave) is changed from 100 to 500 V. Particle chains are successfully pulled out of the liquid when U > 200 V. At high voltages, the particles tend to agglomerate. See also corresponding Movie S1; (**c**) Experimental realization of granular particle chain formed at U = 250 V. Firstly, a signal electrode is dipped into the silicone oil dispersion. The PS particles (sulfonated for 32 min) move towards the electrode by DEP force. The electrode is then lifted and the particles follow the electrode, eventually forming a 1D structure outside the liquid environment. See also corresponding Movie S4.

A schematic representation of the fabrication process of granular chains is shown in Figure 4a. A signal electrode is initially dipped in a dispersion of silicone oil and PS microparticles, which are settled on a grounded conductive plate. When an AC voltage is applied over the signal electrode and conductive plate, the dispersed particles move towards the electrode by dielectrophoretic (DEP) force. The electrode is then lifted and the particles move upwards, following the electrode motion. When a particle is lifted to the liquid-air interface, a capillary bridge is formed between the particle and the planar interface (hereafter referred to as capillary bridge “type I” to distinguish it from the capillary bridge “type II” that is formed between two particles after a particle has been lifted above the liquid-air interface). The capillary bridge (type I) tends to pull the particle back to the liquid. To successfully pull the particle outside the liquid, the DEP force acting on that particle has to be larger than the oppositely working capillary force owing to the presence of the capillary bridge (type I).

The downward capillary force stemming from a liquid capillary bridge (type I) formed between a spherical particle of radius R and a planar liquid interface with surface tension γ is given by: $F_{\mathrm{sp}}\approx 2\pi \gamma R$. The DEP force between two particles is given by the standard DEP model [48]: $F_{\mathrm{DEP}}=8\pi \varepsilon_{\mathrm{oil}}\varepsilon_{0}R^{5}\beta U^{2}/{\left(2R+s\right)}^{5},$ where *s* is the distance between spherical particles (or a spherical particle and an electrode that is here approximated to have the same shape and size as the spherical particle). When two spherical particles are in contact (*s*→0), the applied voltage U should be at least $U_{min}=\sqrt{8\gamma R/\varepsilon_{\mathrm{oil}}\varepsilon_{0}\beta }$ to generate a force sufficiently large to pull the particle outside the liquid-air interface. Given that γ ~20 mN/m, R ~70 μm, $\varepsilon_{\mathrm{oil}}$ ~2.8, and β ~0.1, the minimum voltage required for a particle to be removed from the liquid interface is around 700 V. Based on our previous experimental and theoretical work [29], we know that the conventional DEP model significantly underestimates the magnitude of the DEP force, and thus we expect $U_{min}$ to be around 200 V for these experiments.

Figure 4b shows experiments on granular particle chain fabrication. In these experiments, we used PS particles sulfonated for 32 min dispersed in silicone oil, and we changed the strength of the applied AC voltage (*f* = 100 Hz, square wave) from 100 V to 500 V. For electric voltages below 200 V, it was not possible to form chains. Applying a voltage of 200 V, we succeeded five out of 10 times to form short chains (few-particle long chains). At U = 250 V, we succeeded nine out of 10 times to form chains that were on average around one order of magnitude longer than those formed at U = 200 V. A further increase of the applied voltage enabled formation of longer chains with higher success rates. However, at voltages of around 500 V and higher, the PS particles tended to agglomerate, as demonstrated in the right panel of Figure 4b. Such agglomerated structures are typically unstable when the electric field is turned off, whereas chains with a one-particle length are stable in the absence of an electric field. The stability of one-particle long chains is owed to the attractive interaction of the capillary bridges (type II) between two neighboring particles formed from the capillary bridges (type I) just after a particle has passed the liquid-air interface (for details regarding the formation of capillary bridge formation type I and its transition to capillary bridge type II when pulling a chain of spherical particles outside the liquid, we refer to the following research article: [1]).

PS particles sulfonated for 4, 8, and 16 min exhibit similar behavior to changes in applied voltage as the particles sulfonated for 32 min, as presented in Figure 4b. For PS particles sulfonated for 2 min, higher voltages are required to pull particle structures out of the liquid. However, the success rate for forming 1D chains with PS particles sulfonated for 2 min is significantly lower than particles sulfonated for longer times. At voltages between 300 V and 400 V, the PS particles sulfonated for 2 min can only form short chains, and at higher voltages, they form short agglomerated structures (see corresponding Movie S2). For PS particles sulfonated for 1 min, we were not able to form single-particle long 1D structures, nor agglomerated structures (see also Movie S3). A column chart of particle chain length versus applied electric voltage and particle sulfonation time is presented in Figure S1. The chart shows that PS particles sulfonated for 32 min consistently forms longer chains than PS particles sulfonated for 2 min.

Figure 4c presents the successful experimental realization of a granular particle chain. First, a signal electrode at potential of 250 V and frequency of 100 Hz is dipped into a silicone oil dispersion. The PS particles (sulfonated for 32 min) move towards the electrode by DEP force. The electrode is then lifted (t > 1 s) and the particles follow the electrode, eventually forming a 1D granular particle structure outside the liquid environment (see also Movie S4).

**Figure 5:**
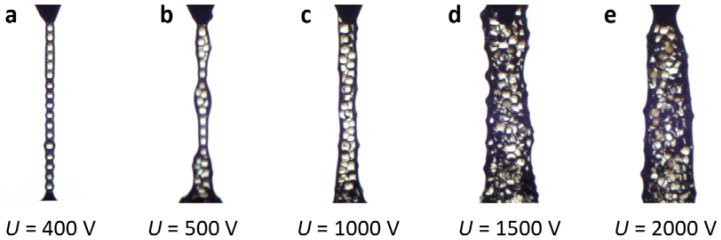
1D structures made of aggregated PS particles sulfonated for 32 min. The structures were formed by pulling the particles out of silicone oil with a velocity of ~1 mm/s to a height of ~2.5 mm applying an electric voltage of (**a**) 400 V; (**b**) 500 V; (**c**) 1000 V; (**d**) 1500 V; and (**e**) 2000 V.

As mentioned above, when applying high voltages (>500 V), the PS particles tend to agglomerate. We exploit this to produce 1D structures composed of aggregated particles. In Figure 5, we demonstrate five structures of different thicknesses (single particle-thick to around 5 particles-thick) by varying the applied electric voltage from 400 V to 2000 V. The structures length is around 2.5 mm, and the signal electrode was pulled up from the dispersion with a velocity of ~1 mm/s.

**Figure 6:**
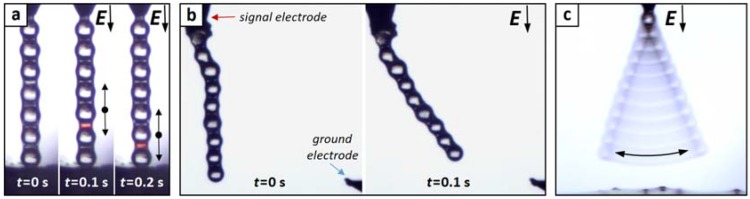
(**a**) Demonstration of contact-charge electrophoresis. A particle chain is placed between the signal electrode and a grounded plate. The distance between the electrodes is larger (around a one-particle radius) than the sum of the particle diameters comprising the chain. This distance enables particle motion along the chain direction. A DC electric field is turned on (t > 0 s) and some of the particles oscillate up and down, as indicated by the arrows. Large gaps between particles are marked with red color. See also corresponding Movie S5; (**b**) Active bending of a particle chain. When a DC electric field is applied, the chain bends towards the ground electrode. The bending is permitted due to the chain flexibility caused by liquid bridges between particles. The chain movement is reversible by turning off the electric field. See also corresponding Movie S6; (**c**) Oscillation of a granular chain. The particle chain oscillates with a frequency of more than 25 Hz when subjected to a voltage around 300 V and frequency of 50 Hz. The oscillations stop after increasing the frequency to 200 Hz. See also corresponding Movie S7.

Employing DC electric fields introduces some physical phenomena that may disrupt chain formation, including the induction of electrohydrodynamic liquid flows [49], or contact-charge electrophoresis (CCEP) [50]. The latter is demonstrated in Figure 6a and in supplementary Movie S5. Both show the standard CCEP oscillation occurring at low frequencies, a phenomenon that destabilizes formed particle chains, and may also cause chains to break during their formation, which in turn may reduce the success rate of pulling particle chains outside the liquid. This problem can be resolved by utilizing high-frequency AC fields, which quickly desynchronize the charges on the particle spheres and thus largely suppress CCEP. In Movie S5, 100 Hz is sufficient to significantly reduce the CCEP effect. On the other hand, by increasing the frequency of the electric field, the dielectric constant of particles decreases (as demonstrated in Figure 1a) and the attractive electrostatic interaction decreases. Based on the chain length versus frequency plot presented in Figure S2, we suggest the use of a frequency of around 100 Hz to form stable and long particle chains.

As already noted, a particle chain composed of several microparticles remains stable even after the applied voltage is turned off. This is possible as long as the capillary force between particles—originating from capillary bridges (type II)—is larger than the gravitational force acting on the entire microparticle chain. In Figure 6b, we demonstrate that the formed particle chain can be actively bent by switching the electric field on and off. In the absence of an electric field, the PS particle chain attached to the signal electrode (top left corner of the image), rests in a vertical direction, only affected by gravity and capillary bridges (type II). The ground electrode (bottom right corner of the image) was placed away from the particle chain to produce an electric field in a direction 45° from the downward direction of gravity. When the electric field was turned on, the chain bent towards the ground electrode. The chain could bend due to the chain flexibility caused by the liquid bridges between the particles (type II). The chain movement is reversible by turning off the electric field, and the bending angle of the chain can be controlled by tuning the voltage strength, as demonstrated in Movie S6.

The particle chain stabilized by the capillary liquid bridges shows remarkable mechanical strength. In Figure 6c and the corresponding Movie S7, a particle chain composed of PS particles oscillates with a frequency larger than 25 Hz (from the 25 fps movie recording, we cannot estimate the exact frequency). The assembled particle chain was brought near the surface of the dispersion (around one particle away from the surface) and a voltage of around 300 V and a frequency of 50 Hz was applied. The oscillations terminated when the frequency was increased above 100 Hz.

## 3. Discussion and Conclusions

Being able to tailor the electric properties of nano- and micro-sized particles allows for numerous applications within material development. In this work, we demonstrate that sulfonation, a cost-efficient method, can be used to modify the electric properties of polystyrene particles by just adjusting the sulfonation reaction time. Dielectric spectroscopy measurements show that both the electrical conductivity and dielectric constant values of the polystyrene particles increase with longer sulfonation time. This result is expected because the degree of sulfonation increases with reaction time [51], allowing more water to adsorb to the PS particles [45,52] and thus enhance protonic conductivity [53]. In our previous research [42], we validated the influence of water content on the dielectric properties of sulfonated PS particles. The effect of water content on dielectric properties and the electrorheological response of sulfonated polystyrene samples was also studied in Reference [54]. By increasing the water content, the authors of Reference [54] experimentally observed an increase in dielectric constant and electrical conductivity values, as well as a frequency shift in the dissipation factor (the frequency for maximum dielectric energy loss is shifted). Our dielectric spectroscopy measurements also reveal such a frequency shift where the maximum dielectric energy loss is shifted towards higher frequencies for PS particles sulfonated at longer times. The result may indicate an increased amount of adsorbed water at the interface of the particles sulfonated for longer times.

The properties of the modified particles are further investigated by dipolar force measurements performed on particle pairs that are suspended in castor oil and subjected to high frequency (100 Hz) AC electric fields. PS particles sulfonated for longer times exhibit larger attraction force, resulting in higher particle speeds and reduced time taken to approach each other. From measuring the particle speed as a function of particle separation distance, we can estimate the dielectric constant of the particles by fitting the data to the standard dipolar model [47]. The estimated dielectric values for the PS particles attained from the fitting are slightly higher than those we measured by dielectric spectroscopy. These differences in estimated and measured dielectric constant values are expected, since the dielectric spectroscopy measurements were performed on particles in powder form placed in a sample cell containing air. The air between the particles lower the measured electric properties of the powder.

Both the dielectric spectroscopy measurements and the estimation from the particle speed experiments show that the dielectric constant of the PS particles increase with sulfonation time, and that a saturated dielectric constant value is obtain after 8 min of sulfonation. 

In the last part of the Results section, we demonstrate the importance of the particles’ electric properties in forming 1D structures. In our setup, PS particles with low dielectric constant values (non-modified or particles sulfonated for a few minutes) cannot be used to fabricate chains outside liquid environments, even if the applied voltage is high (1000 V). To pull out chains from the liquid, the DEP force pulling the particles together needs to be stronger than the opposing capillary force, stemming from the formation of capillary bridges [29]. PS particles with higher dielectric constant values (sulfonated for more than 8 min) experience a stronger attractive DEP force, allowing particle chains to be pulled out of the liquid applying moderate (250–500 V) voltages. In addition, we presented an approach to fabricating 1D structures made of aggregated PS particles. The thickness of such columnar structures can be controlled by electric voltage (high electric voltages are needed, 500–2000 V). We speculate that electrowetting plays a role in the formation of columnar structures, i.e., at higher electric fields, more liquid is attracted towards the electrode, which affects the shape of the sphere-planar surface capillary bridge. This, in turn, enables pulling out a larger number of particles, that are now more attracted towards the electrode by the strong dielectrophoretic force. The aggregation of PS at the interface during pulling can be a problem when producing long and single-particle-thick chains. Because the surface tension pulls the particles downward with a force proportional to the particle aggregate radius, the pulled chain breaks when the aggregate is large. To form long, stable particle chains, the breakup of chains due to particle aggregation is a larger problem than the DC effects presented in Figure 6a.

We also demonstrate how such particle chains can be bent, oscillate, and bounce by controlling the applied voltage and frequency. Controlling the dynamics of particle chains and the particles within the chains have many applications, including propulsion of nano-robots and biological material [55], and as acoustic switches and logic elements [56]. Such particle assemblies can also be used as a model system, i.e., to study analogous molecular systems [57], or to represent soft dissipative objects [58]. We expect that our findings will encourage further studies on particle modification for tailored granular or colloidal structures, as well as the further development of applications using micro- and nanoparticles as building blocks. Particles with tailored electric properties placed on droplet interfaces could also experience the “pupil effect” reported in Reference [59], and be used to fabricate new materials with advanced optical and material properties. Future research should investigate the effect of electrode tip geometry and applied frequency on chain formation, and how different cations, particle size, strength of acid, and temperatures affect the sulfonation process and resulting particle properties. 

## 4. Materials and Methods

### 4.1. Sulfonation of PS Particles

PS particles (Dynoseeds TS40 6317) with mean diameters of around 140 μm, a density of around 1.05 g·cm^−3^, and without cross-linking were purchased from Microbeads AS, Skedsmokorset, Norway. The general preparation route for the chemical modification of PS particles was as follows: Firstly, sulfuric acid (30 mL, 96% for analysis, 7664-93-9, MerckKGaA, Darmstadt, Germany) was poured into a three-necked round flask and heated up to 50 °C under magnetic stirring and a continuous N_2_ flow in a preheated oil bath. Then, 5 g of the 140 μm PS particles were poured directly from a glass test tube into the sulfuric acid to assure that all the PS particles were fully wetted by the sulfuric acid and sulfonated. After the respective stirring time performed under a continuous N_2_ flow, the sulfonation reaction was ceased using a copious amount of Milli-Q (18 Ω) ice-water. Subsequently, the dispersion was poured into a sintered glass funnel (connected to a side-arm flask with a tube leading to a vacuum pump), and the sulfonated polystyrene particles were rinsed thoroughly with Milli-Q water. Then, the particles were dispersed in 20 ml of 0.2 M KOH solution, re-filtered, and washed with Milli-Q water again. Finally, the sulfonated particles were dried at 75 °C for 24 h. To study the influence of the reaction time on the final physical properties of the sulfonated particles, we made six samples prepared at incremental reaction times: 1, 2, 4, 8, 16, and 32 min, respectively.

### 4.2. Measurements of Dielectric Constant and Electric Conductivity

To determine the dielectric constant and the electrical conductance of the powder samples, we carried out capacitance measurements using an LCR meter (E4980A, Agilent Technologies, Santa Clara, CA, USA) at a frequency (*f*) range between 100 Hz and 100 kHz. A home-made cylindrical capacitor was used as a sample holder, where the separation distance (*l*) between the inner and outer cylindrical electrode was 1.4 mm, and the capacitor (*C*_o_) was measured to be 2.78 pF. The samples were poured into the cylindrical gap, while homogenous filling was provided by shaking the capacitor. We performed measurements with an effective voltage of 1 V at room temperature (24 °C) and at ambient pressure. The LCR meter was set to parallel mode, where the capacity (*C*) and the dissipation factor (tan*δ*) were recorded. Then, the dielectric constant (*ε*) and the electrical conductivity (*σ*) values were calculated by: *ε* = *C*/*C*_o_, *σ* = 2π*fC*tan*δ*(*l*/*A*). 

The fitting of the experimental dielectric constant data was done by employing MATLAB [60]. Because the presented dielectric dispersion resembles a Debye-like relaxation process [61], the well-known empirical Havriliak-Negami (HN) function was applied to fit the data in the frequencies above 100 Hz. The complex fitting function was complemented by the Jonscher’s power law to fit the low frequency asymptotic form of the electrode polarization. Thus, the applied fitting function is represented by the superposition of both HN and Jonscher’s terms, as follows [61]:

(1) $$\varepsilon^{*}\left(\omega \right)=\varepsilon_{\infty }+\frac{\varepsilon_{S}-\varepsilon_{\infty }}{{\left[1+{\left(i\omega \tau \right)}^{\alpha }\right]}^{\beta }}+A{\left(i\omega \right)}^{m},$$

where $\varepsilon_{\infty }$ and $\varepsilon_{S}$ are the high and low frequency limit of the complex dielectric permittivity $\varepsilon^{*}\left(\omega \right)$, τ is the relaxation time of the interfacial polarization, α and β are empirical exponents, *A* is the amplitude, and m is the Jonscher’s stretch parameter. These two terms fit the experimental data well, as depicted in Figure 2a.

### 4.3. Measurement of Dipolar Forces

Sulfonated polystyrene particles were mixed with 100 mPa·s silicone oil and suspended in the same type of oil in a sample cell. The experimental setup used for measuring attractive interactions between pairs of PS particles was similar to that described in Reference [62], except that here the sample cell was made out of glass, and copper electrodes with a size of 10 mm × 50 mm were placed inside the cell and spaced by 18 mm. Using a mechanical pipette with a small pipette tip, the particles were brought in close proximity and aligned along the electric field direction before an AC electric field of 75 V/mm and 100 Hz was applied across the sample cell in the horizontal direction. Movies (25 frames/s) of the particle dynamics were recorded by a CMOS camera (UI-3590CP-C-HQ, IDS Imaging Development Systems GmbH, Obersulm, Germany) mounted on a high-magnification zoom lens system (MVL12X3Z, Thorlabs, Inc., Newton, NJ, USA). The movies were converted into images using Adobe Premiere Pro CC 2017 (2017.1.1, Adobe Systems, San Jose, CA, USA) and Free Video to JPG Converter by DVDVideoSoft (5.0.101, Digital Wave Ltd., London, UK). From each image, the particle separation distance, radius, speed, and acceleration were measured and calculated in the software ImageJ (1.5.1j8, open source software, National Institutes of Health, Bethesda, MD, USA). The measured particle speed and separation distance *s* of each image were averaged to every 10- pixel, i.e., so that *s* goes from maximum to 0 (particles in contact) in steps of 10 pixels. Finally, the particle speed and separation distance of several measurements (using same particle type and applied electric field) were averaged. 

### 4.4. Experimental Setup for 1D Particle Assembly

The experimental setup for the electric field-driven particle assembly of PS particles consisted of a signal electrode made of a stainless steel medical needle mounted on a mechanical x-y-z translational stage, a grounded conductive plate placed on another mechanical x-y-z translational stage, a CMOS camera (UI-3590CP-C-HQ, IDS Imaging Development Systems GmbH, Obersulm, Germany) mounted on a high-magnification zoom lens system (MVL12X3Z, Thorlabs, Inc., Newton, NJ, USA), a signal generator, a voltage amplifier, and a PC for recording images and movies. The high-voltage electric signal was obtained by amplifying a low-voltage signal (SDG1025, SIGLENT Technologies America, Inc., Solon, OH, USA) using a high-voltage bipolar amplifier (10HVA24-BP1 HVP High Voltage Products GmbH, Munich, Germany). The AC electric signal was always square-shaped and bipolar, and its value was provided as the root-mean-square (RMS) value. Silicone oil (200/350 mPa·s, Dow Corning, Auburn, AL, USA, electrical conductivity approximately 3–5 pS·m^−1^, relative permittivity 2.8) droplets containing sulfonated PS particles were introduced onto the conductive plate. A needle-shaped electrode was dipped into the dispersion and, after the voltage was applied, the electrode was slowly lifted up to pull the PS particles out of the dispersion.

## Figures and Tables

**Table 1:** List of particle dielectric constant (ε) and electrical conductivity (*σ*) values measured at a frequency of 100 Hz, and dielectric strength ($\varepsilon_{S}-\varepsilon_{\infty }$) values estimated from fitting the dielectric constant experimental data with theoretical models.

Sulfonation Time (min)	0	1	2	4	8	16	32
Dielectric constant, ε	2.2	2.7	2.9	3.2	6.3	7.4	7.9
Electrical conductivity, *σ* (nS/m)	0.5	4.5	8.1	43	81	81	83
Dielectric strength ($\varepsilon_{S}-\varepsilon_{\infty }$)	0.7	0.8	0.9	1.1	4.1	4.3	4.5

**Table 2:** Values of the estimated dielectric mismatch (β) and particle dielectric constant ($\varepsilon_{p}$).

Sulfonation Time (min)	1	2	4	8	16	32
Estimated β	0.01	0.23	0.37	0.38	0.41	0.36
Estimated $\varepsilon_{p}$	3.5	5.3	7.6	7.9	8.5	7.6

## Supplementary Materials

The following are available online at www.mdpi.com/1996-1944/10/10/1212/s1: Movie S1. Experiments on granular particle chain fabrication using PS particles modified for 32 min. The strength of the applied voltage (f = 100 Hz, square wave) is changed from 100 V to 500 V. Particle chains are successfully pulled out of the liquid when U > 200 V. At high voltages, the particles tend to agglomerate; Movie S2. Experiments on granular particle chain fabrication using PS particles modified for 2 min. The strength of the applied voltage (f=100 Hz, square wave) is increased from 100 V to 500 V. Only short agglomerated structures can be formed at high voltages; Movie S3. Experiments on granular particle chain fabrication using PS particles modified for 1 min. The strength of the applied voltage (f=100 Hz, square wave) is increased from 100 V to 500 V. It is difficult if not impossible to form single-particle-long 1D structures or agglomerated structures; Movie S4. Experimental realization of granular particle chain. A signal electrode at a potential of 250 V is dipped into the silicone oil dispersion. The PS particles (sulfonated for 32 min) move towards the electrode by DEP force. The electrode is then lifted up and the particles follow the electrode, eventually forming a 1D structure outside the liquid environment; Movie S5. Contact charge electrophoretic particle oscillations. A granular chain composed of 140-µm PS32 particles is assembled in air. The chain was initially pulled out from silicone oil and then placed between the signal electrode and grounded plate. To enable particle motion along the chain direction, the distance between the electrodes was set to around a one-particle radius larger than the sum of the particle diameters. After the DC electric field is turned on (t > 0 s), some of the particles oscillate up and down. Silicone oil capillary bridges (type II) are clearly resolved in the movie. At low voltage frequencies (below~100 Hz), the particles in the chain move back and forth towards the electrode; Movie S6. Active bending of the particle chain. When the electric field was turned on, the chain bent towards the ground electrode. The bending was permitted due to the chain flexibility caused by the liquid bridges between particles (type II); Movie S7. Oscillation of the granular chain. The particle chain oscillates with a frequency of at least 25 Hz when the applied voltage was around 300 V, 50 Hz. The oscillations stopped after changing the frequency to 200 Hz; Figure S1. Column charts showing maximal chain lengths in 10 experiments for PS particles sulfonated for 2 and 32 min; Figure S2. Maximal chain length versus frequency of applied electric voltage.

Supplementary File 2

Supplementary File 3

Supplementary File 4

Supplementary File 5

Supplementary File 6

Supplementary File 7

Supplementary File 8

## References

[1] Dutka F., Rozynek Z., Napiórkowski M. (2017). Continuous and discontinuous transitions between two types of capillary bridges on a beaded chain pulled out from a liquid. Soft Matter.

[2] Xu K., Qin L., Heath J.R. (2009). The crossover from two dimensions to one dimension in granular electronic materials. Nat. Nanotechnol..

[3] Stephenson C., Hubler A. (2015). Stability and conductivity of self assembled wires in a transverse electric field. Sci. Rep..

[4] Quinten M., Leitner A., Krenn J.R., Aussenegg F.R. (1998). Electromagnetic energy transport via linear chains of silver nanoparticles. Opt. Lett..

[5] Solis D., Willingham B., Nauert S.L., Slaughter L.S., Olson J., Swanglap P., Paul A., Chang W.-S., Link S. (2012). Electromagnetic energy transport in nanoparticle chains via dark plasmon modes. Nano Lett..

[6] Tang L., Yu G., Li X., Chang F., Zhong C.-J. (2015). Palladium-gold alloy nanowire-structured interface for hydrogen sensing. Chempluschem.

[7] Karg M., König T.A.F., Retsch M., Stelling C., Reichstein P.M., Honold T., Thelakkat M., Fery A. (2015). Colloidal self-assembly concepts for light management in photovoltaics. Mater. Today.

[8] Su M., Li F., Chen S., Huang Z., Qin M., Li W., Zhang X., Song Y. (2016). Nanoparticle based curve arrays for multirecognition flexible electronics. Adv. Mater..

[9] Shen S.C., Liu W.-T., Diao J.-J. (2015). Colloidally deposited nanoparticle wires for biophysical detection. Chin. Phys. B.

[10] Li F., Badel X., Linnros J., Wiley J.B. (2005). Fabrication of colloidal crystals with tubular-like packings. J. Am. Chem. Soc..

[11] Rozynek Z., Wang B., Fossum J.O., Knudsen K.D. (2012). Dipolar structuring of organically modified fluorohectorite clay particles. Eur. Phys. J. E.

[12] Li C., Tan J., Li H., Gu J., Zhang B., Zhang Q. (2015). Fast magnetic-field-induced formation of one-dimensional structured chain-like materials via sintering of Fe_3_O_4_/poly(styrene-co-n-butyl acrylate-co-acrylic acid) hybrid microspheres. RSC Adv..

[13] Bharti B., Findenegg G.H., Velev O.D. (2012). Co-assembly of oppositely charged particles into linear clusters and chains of controllable length. Sci. Rep..

[14] Jiang L., Chen X., Lu N., Chi L. (2014). Spatially confined assembly of nanoparticles. Acc. Chem. Res..

[15] Breidenich J.L., Wei M.C., Clatterbaugh G.V., Benkoski J.J., Keng P.Y., Pyun J. (2012). Controlling length and areal density of artificial cilia through the dipolar assembly of ferromagnetic nanoparticles. Soft Matter.

[16] Vilfan M., Potocnik A., Kavcic B., Osterman N., Poberaj I., Vilfan A., Babic D. (2010). Self-assembled artificial cilia. Proc. Natl. Acad. Sci. USA.

[17] Hill L.J., Pyun J. (2014). Colloidal polymers via dipolar assembly of magnetic nanoparticle monomers. ACS Appl. Mater. Interface.

[18] Endo H., Mochizuki Y., Tamura M., Kawai T. (2013). Fabrication and functionalization of periodically aligned metallic nanocup arrays using colloidal lithography with a sinusoidally wrinkled substrate. Langmuir.

[19] Hornyak G., Kroll M., Pugin R., Sawitowski T., Schmid G., Bovin J.O., Karsson G., Hofmeister H., Hopfe S. (1997). Gold clusters and colloids in alumina nanotubes. Chem. Eur. J..

[20] Huang J., Tao A.R., Connor S., He R., Yang P. (2006). A general method for assembling single colloidal particle lines. Nano Lett..

[21] Favier F., Walter E.C., Zach M.P., Benter T., Penner R.M. (2001). Hydrogen sensors and switches from electrodeposited palladium mesowire arrays. Science.

[22] Bharti B., Velev O.D. (2015). Multidirectional, multicomponent electric field driven assembly of complex colloidal chains. Z. Phys. Chem..

[23] Vutukuri H.R., Demirors A.F., Peng B., van Oostrum P.D.J., Imhof A., van Blaaderen A. (2012). Colloidal analogues of charged and uncharged polymer chains with tunable stiffness. Angew. Chem..

[24] Gangwal S., Pawar A., Kretzschmar I., Velev O.D. (2010). Programmed assembly of metallodielectric patchy particles in external ac electric fields. Soft Matter.

[25] Ding H., Liu W., Ding Y., Shao J., Zhang L., Liu P., Liu H. (2015). Particle clustering during pearl chain formation in a conductive-island based dielectrophoretic assembly system. RSC Adv..

[26] Fossum J.O., Meheust Y., Parmar K.P.S., Knudsen K.D., Maloy K.J., Fonseca D.M. (2006). Intercalation-enhanced electric polarization and chain formation of nano-layered particles. EPL.

[27] Xie Q., Davies G.B., Harting J. (2016). Controlled capillary assembly of magnetic Janus particles at fluid-fluid interfaces. Soft Matter.

[28] Kokot G., Piet D., Whitesides G.M., Aranson I.S., Snezhko A. (2015). Emergence of reconfigurable wires and spinners via dynamic self-assembly. Sci. Rep..

[29] Rozynek Z., Han M., Dutka F., Garstecki P., Józefczak A., Luijten E. (2017). Formation of printable granular and colloidal chains through capillary effects and dielectrophoresis. Nat. Commun..

[30] Lascelles S.F., Armes S.P. (1997). Synthesis and characterization of micrometre-sized, polypyrrole-coated polystyrene latexes. J. Mater. Chem..

[31] Yan J., Wang C., Gao Y., Zheng Z., Zhong S., Miao X., Cui X., Wang H. (2011). Anchoring conductive polyaniline on the surface of expandable polystyrene beads by swelling-based and in situ polymerization of aniline method. Chem. Eng. J..

[32] Kim Y., Park D. (2002). The electrorheological responses of suspensions of polypyrrole-coated polyethylene particles. Colloid Polym. Sci..

[33] Han M.G., Sperry J., Gupta A., Huebner C.F., Ingram S.T., Foulger S.H. (2007). Polyaniline coated poly(butyl methacrylate) core-shell particles: Roll-to-Roll printing of templated electrically conductive structures. J. Mater. Chem..

[34] Brijmohan S.B., Shaw M.T. (2006). Proton exchange membranes based on sulfonated crosslinked polystyrene micro particles dispersed in poly(dimethyl siloxane). Polymer.

[35] Kim J.Y., Kwon S., Ihm D. (2004). Reliability and thermodynamic studies of an anisotropic conductive adhesive film (ACAF) prepared from epoxy/rubber resins. J. Mater. Process. Technol..

[36] Yuan Y., Lian Y. (2009). Polystyrene microspheres coated with smooth polyaniline shells: Preparation and characterization. Tsinghua Sci. Technol..

[37] Kubarkov A.V., Pyshkina O.A., Karpushkin E.A., Stevenson K.J., Sergeyev V.G. (2017). Electrically conducting polymeric microspheres comprised of sulfonated polystyrene cores coated with poly(3,4-ethylenedioxythiophene). Colloid Polym. Sci..

[38] Piao S.H., Gao C.Y., Choi H.J. (2017). Sulfonated polystyrene nanoparticles coated with conducting polyaniline and their electro-responsive suspension characteristics under electric fields. Polymer.

[39] Fan W., Zhang C., Tjiu W.W., Liu T.X. (2013). Fabrication of electrically conductive graphene/polystyrene composites via a combination of latex and layer-by-layer assembly approaches. J. Mater. Res..

[40] Pan Y.F., Wang J.N., Wang Y., Wang Z.Q. (2014). PS microspheres coated by AuNPs via thermodynamic driving heterocoagulation and their high catalytic activity. Macromol. Rapid. Commun..

[41] Lee J.-H., Lee Y., Nam J.-D. (2009). Tunable surface metal morphologies and electrical properties of monodispersed polystyrene beads coated with metal multilayers via electroless deposition. Intermetallics.

[42] Mikkelsen A., Wojciechowski J., Rajňák M., Kurimský J., Khobaib K., Kertmen A., Rozynek Z. (2017). Electric field-driven assembly of sulfonated polystyrene microspheres. Materials.

[43] Asako Y., Ono S., Aizawa R., Kawakami T., Havelka K.O.L., Filisko F.E. (1995). Properties of electrorheological fluids containing numerously sulfonated polymer particles. Progress in Electrorheology: Science and Technology of Electrorheological Materials.

[44] Asako Y., Ono S., Aizawa R., Kawakami T. (1996). Properties of electrorheological fluids containing sulfonated poly(styrene-co-divinylbenzene) particles. Int. J. Mod. Phys. B.

[45] Fan X., Niu L., Wu Y.H., Cheng J., Yang Z.R. (2015). Assembly route toward raspberry-like composite particles and their controlled surface wettability through varied dual-size binary roughness. Appl. Surf. Sci..

[46] Fan X., Niu L., Xia Z. (2014). Preparation of raspberry-like silica microcapsules via sulfonated polystyrene template and aniline medium assembly method. Colloid Polym. Sci..

[47] Davis L.C. (1992). Polarization forces and conductivity effects in electrorheological fluids. J. Appl. Phys..

[48] Jones T.B. (2005). Electromechanics of Particles.

[49] Saville D.A. (1997). Electrohydrodynamics: The taylor-melcher leaky dielectric model. Annu. Rev. Fluid Mech..

[50] Drews A.M., Cartier C.A., Bishop K.J.M. (2015). Contact charge electrophoresis: Experiment and theory. Langmuir.

[51] Kucera F., Jancar J. (1996). Preliminary study of sulfonation of polystyrene by homogeneous and heterogeneous reaction. Chem Pap.

[52] Benavides R., Oenning L.W., Paula M.M.S., Da Silva L. (2014). Properties of polystyrene/acrylic acid membranes after sulphonation reactions. J. New Mat. Electrochem. Syst..

[53] Wallace R.A. (1973). Electrical-conduction in sulfonated polystyrene films. J. Appl. Polym. Sci..

[54] Ikazaki F., Kawai A., Kawakami T., Konishi M., Asako Y. (1999). Electrorheology of suspension of sulfonated styrene-co-divinylbenzene particles - approach based on the dielectric properties of the suspension. Int. J. Mod. Phys. B.

[55] Dreyfus R., Baudry J., Roper M.L., Fermigier M., Stone H.A., Bibette J. (2005). Microscopic artificial swimmers. Nature.

[56] Li F., Anzel P., Yang J., Kevrekidis P.G., Daraio C. (2014). Granular acoustic switches and logic elements. Nat. Commun..

[57] Li D.C., Banon S., Biswal S.L. (2010). Bending dynamics of DNA-linked colloidal particle chains. Soft Matter.

[58] Sun Y.C., Fei H.T., Huang P.C., Juan W.T., Huang J.R., Tsai J.C. (2016). Short granular chain under vibration: Spontaneous switching of states. Phys. Rev. E.

[59] Dommersnes P., Rozynek Z., Mikkelsen A., Castberg R., Kjerstad K., Hersvik K., Otto Fossum J. (2013). Active structuring of colloidal armour on liquid drops. Nat. Commun..

[60] Axelrod N., Axelrod E., Gutina A., Puzenko A., Ben Ishai P., Feldman Y. (2004). Dielectric spectroscopy data treatment: I. Frequency domain. Meas. Sci. Technol..

[61] Kremer F., Schönhals A. (2003). Broadband Dielectric Spectroscopy.

[62] Rozynek Z., Dommersnes P., Mikkelsen A., Michels L., Fossum J.O. (2014). Electrohydrodynamic controlled assembly and fracturing of thin colloidal particle films confined at drop interfaces. Eur. Phys. J.-Spec. Top..

