# Supplementary material for: Assembly of 1D Granular Structures from Sulfonated Polystyrene Microparticles
Source: Materials (Basel). 2017 Oct 21;10(10):1212. doi: 10.3390/ma10101212 (PMC5667018; doi:10.3390/ma10101212)
Supplement: Supplementary file 1 [file materials-10-01212-s001.docx]

Supplementary Materials: Assembly of 1D granular structures from sulfonated polystyrene microparticles

Alexander Mikkelsen ^1^, Ahmet Kertmen ^2,3^, Khobaib Khobaib ^1^, Michal Rajňák ^4,5^, Juraj Kurimský ^5^ and Zbigniew Rozynek ^1,^*


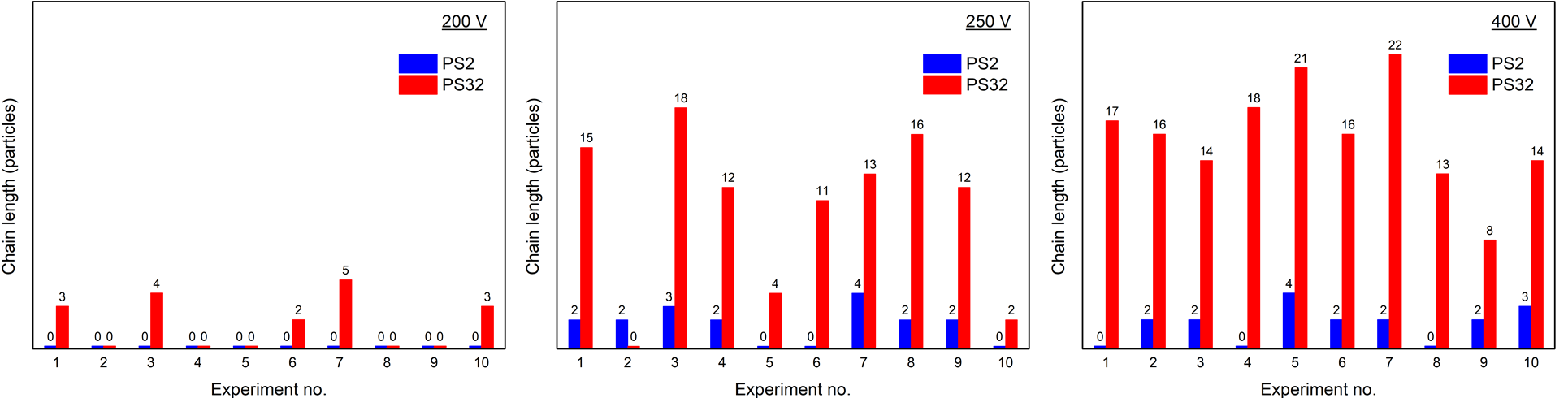


**Figure S1.** Column charts showing maximal chain lengths in ten experiments for polystyrene (PS) particles sulfonated for 2 mins (blue) and 32 min (red). Electric voltage of three different strengths were (frequency of 100 Hz, square wave) applied, namely 200 V, 250 V, and 400 V. The electrode was pulled up from the dispersion at a rate of 10 µm·s^−1^.


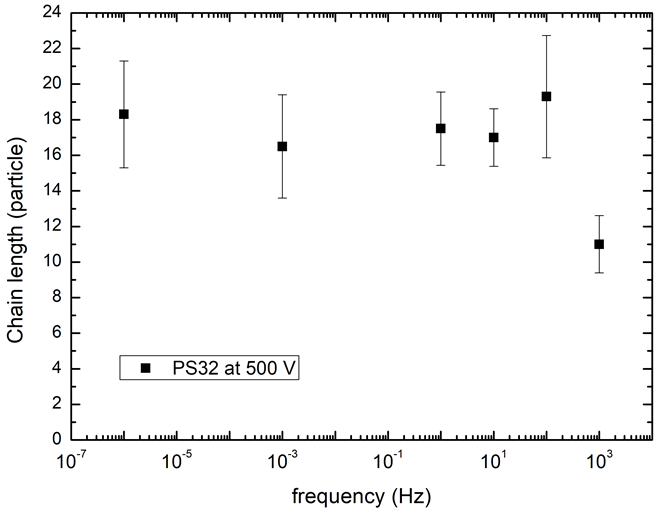


**Figure S2.** Maximal chain length versus frequency of applied electric voltage of 500 V, square wave. The experimental error is calculated as a standard deviation of the results from ten experiments. The electrode was pulled up from the dispersion at rate of 20 µm·s^−1^, and the chains were made of PS particles sulfonated for 32 min. The particle chain length decreases at frequencies >100 Hz. This is in accordance with the frequency spectra presented in the main text (Figure 2a).
